# Editorial: Advances in medical imaging for precision diagnostic and therapeutic applications in digestive diseases

**DOI:** 10.3389/fmed.2026.1835279

**Published:** 2026-05-06

**Authors:** Fu Shen, Feng Shi, Hao Wang, Yiqun Sun, Yichen Ding

**Affiliations:** 1 Changhai Hospital, Naval Medical University, Shanghai, China; 2 Department of Research and Development, United Imaging Intelligence, Shanghai, China; 3 Fudan University Shanghai Cancer Center, Shanghai, China; 4 Department of Bioengineering, The University of Texas at Dallas, Richardson, TX, United States

**Keywords:** artificial intelligence, digestive diseases, gastroenterology, medical imaging, precision medicine, radiomics

Gastroenterology is undergoing a profound transformation, propelled by rapid advances in medical imaging. From multimodal techniques to artificial intelligence (AI)-driven diagnostics, the integration of innovative imaging technologies into clinical practice is redefining how digestive diseases are detected, characterized, and managed (1–7). In this Research Topic, we present a collection of 20 articles that highlight the expanding role of imaging—encompassing MRI, CT, endoscopy, and fusion imaging—in enabling precision diagnosis and guiding targeted therapy. Covering a wide spectrum of gastrointestinal pathologies including hemorrhoids, anal fistulas, colorectal cancer, pancreatitis, esophageal cancer, and gastric disorders, these contributions offer a comprehensive view of current innovations and future directions in digestive disease imaging.

## Imaging-guided precision in surgical and interventional care

Several studies underscore the critical role of preoperative imaging in optimizing surgical outcomes for anorectal disorders. The integration of MRI into preoperative assessment for patients undergoing procedure for prolapse and hemorrhoids (PPH), as demonstrated in a retrospective cohort study by Zhang, Zhao, Wei et al., yielded significant clinical benefits. These benefits included reduced intraoperative blood loss, accelerated recovery, and higher patient satisfaction, particularly when combined with day surgery protocols.

Similarly, in the context of anal fistula excision, a related study by Zhang, Chen Wei et al. found that MRI achieved exceptional accuracy in classifying fistula anatomy according to the Parks classification and identifying the number of fistulous tracts (Kappa value 0.948). This precise preoperative mapping facilitated tailored surgical planning, minimized recurrence risk, and supported efficient day surgery protocols. These findings validate MRI as an indispensable tool for preoperative stratification, enabling minimally invasive, resource-efficient care that shortens hospital stays and aligns with modern healthcare demands. Given its superior soft tissue contrast, MRI is uniquely suited for perianal and pelvic applications. When combined with day surgery frameworks, imaging not only improves clinical outcomes but also optimizes healthcare efficiency by accelerating patient recovery and reducing inpatient burden.

## AI and radiomics: redefining precision in digestive oncology

A compelling theme emerging from this Research Topic is the translation of AI and radiomics from experimental concepts to clinical practice for digestive oncology. These technologies are transforming imaging from a purely anatomical tool into a means of non-invasive molecular and pathological profiling. A standout study by Wang et al. developed a CT-based radiomic model to predict KRAS gene mutation status in colorectal cancer (CRC), creating a “virtual biopsy” alternative to invasive tissue sampling. The XGBoost model achieved high predictive accuracy, with an AUC of 0.90 in the training set and 0.81 in the test set, offering critical genetic information to guide personalized targeted therapy without the risks of physical biopsy.

Complementing this, Wei et al. presented a hybrid model for predicting microsatellite instability (MSI) in CRC. By combining fully-supervised deep learning analysis of hematoxylin & eosin-stained images with clinical features, the model achieved an AUC of 0.949 in internal validation and 0.862 in external testing. Identification of hub genes (IFNG, CD8A) and enriched immune pathways further links imaging-derived phenotypes to molecular biology, deepening understanding of CRC pathogenesis.

Furthermore, bibliometric analyses, such as the comprehensive study by Letafatkar et al., document explosive growth in AI research for endoscopy and colonoscopy, focusing primarily on real-time applications such as adenoma detection and polyp segmentation. Corroborating this, an umbrella review by Huang et al., which confirmed AI's high diagnostic performance for early cancers and precancerous lesions, often outperforming expert endoscopists, while noting that video-based AI models require further refinement. Collectively, these studies establish AI-imaging integration as a cornerstone of next-generation precision oncology.

## Endoscopic innovation: enhancing diagnostic accuracy and patient safety

Endoscopic techniques continue to evolve, with several studies highlighting novel approaches to improve diagnostic precision and safety, particularly for high-risk patients. The application of detachable string magnetically controlled capsule endoscopy (ds-MCE) in patients on antithrombotic therapy represents a major advance in non-invasive gastrointestinal visualization. As demonstrated in a pilot study by Bai et al., compared to conventional wireless capsule endoscopy, ds-MCE achieved markedly superior visualization of the esophageal mucosa and Z-line (52.3% complete visualization vs. 6.3%), with high tolerability and no capsule retention. This modality offers a valuable non-invasive alternative to esophagogastroduodenoscopy for this vulnerable group facing elevated bleeding risks.

For superficial esophageal squamous cell carcinoma, Zhang, Chen, Gao et al. found that endoscopic ultrasound with submucosal saline injection (EUS-SSI) significantly improved T1a/T1b staging accuracy (84.8% vs. 68.5% with conventional EUS). By mitigating over-staging, this simple procedural refinement ensures appropriate treatment selection—whether endoscopic resection or radical surgery—avoiding unnecessary invasive interventions. Additionally, a multicenter study by Wu, Tan et al. evaluating blue laser imaging (BLI) combined with the JNET classification for colorectal sessile lesions found that expert endoscopists achieved 93.1% accuracy for low-grade dysplasia. While endoscopist experience remains critical, these findings emphasize the value of standardized classification systems in supporting less experienced clinicians and ensuring diagnostic consistency.

## Multimodal imaging: synergizing strengths for complex diagnoses

The enhanced diagnostic power of multimodal imaging fusion emerges as a recurring theme. Exemplifying this approach, a case report by Wu, Gao et al. on contrast-enhanced ultrasound (CEUS)/CT fusion navigation for detecting hidden peripancreatic abscesses in acute pancreatitis exemplifies this approach, combining real-time ultrasound with detailed CT anatomical mapping to precisely localize lesions and guide interventional drainage with improved accuracy and safety. Such fusion strategies are poised for wider adoption as software integration advances.

Furthermore, a comparative analysis by Zhao et al. of CT and MRI in preoperative TNM staging of esophageal cancer confirms both modalities deliver high diagnostic value. CT remains the more accessible and cost-effective option for routine staging, while MRI's superior soft tissue contrast and multiplanar capabilities make it indispensable for evaluating T4 disease and tumor invasion of adjacent organs. The study also identifies persistent limitations in early T- and N-stage assessment, pointing to the need for multimodal strategies and targeted contrast agents to further enhance staging precision.

## Unresolved challenges and critical clinical considerations

Despite significant advances, several studies highlight ongoing challenges. A systematic review and meta-analysis by Li, Li et al. comparing [68Ga]Ga-FAPI-04 PET and [18F]FDG PET for detecting lymph node metastasis in digestive system cancers found the former offered significantly higher sensitivity (0.82 vs. 0.51) with comparable specificity, yet substantial study heterogeneity warrants cautious interpretation and underscores the need for large prospective trials.

The impact of anesthesia on colonoscopy quality metrics also emerged as another key consideration. A large retrospective study of over 12,000 colonoscopy cases by Cai et al. found non-anesthesia-assisted colonoscopy was associated with a significantly higher adenoma detection rate (36.94% vs. 26.40%), highlighting the delicate balance between patient comfort and diagnostic thoroughness. Additionally, a retrospective cohort study of 318 patients by Li, Lu et al. identified key risk factors for colorectal polyp recurrence—including male gender, age 60–80 years, gallbladder disease, food allergy, adenomatous pathology, and Helicobacter pylori infection—with larger polyp size (≥2 cm) acting as a protective factor in their cohort. The resulting prediction model (sensitivity 0.88, specificity 0.56) provides a practical tool for identifying high-risk patients requiring more intensive post-surgical surveillance.

## Future directions and conclusion

The contributions to this Research Topic collectively illustrate a field in dynamic evolution, where medical imaging has evolved from a diagnostic adjunct into an integral component of therapeutic planning, risk stratification, and outcome prediction. The integration of AI, multimodal fusion, and functional imaging is paving the way for more precise, personalized, and minimally invasive care.

To realize the full potential of these advances requires addressing several priorities. Large prospective trials are needed to validate novel modalities such as [68Ga]Ga-FAPI-04 PET and ds-MCE. AI models require further refinement—particularly for video applications—and external validation across diverse populations. Integrating imaging with multi-omics data holds promise for developing even more personalized diagnostic and therapeutic models. Ongoing research into imaging-guided minimally invasive interventions for complex conditions like necrotizing pancreatitis and refractory esophageal stricture is essential. Finally, standardizing imaging classification systems and implementing targeted clinician training will be critical to translating technological advances into consistent, high-quality care.

In summary, the studies in this Research Topic exemplify remarkable progress in medical imaging for digestive diseases, showcasing how innovations in imaging modalities, AI integration, and endoscopic technology are advancing precision diagnosis and therapy. As healthcare shifts toward personalized, value-based care, medical imaging will remain at the forefront, empowering clinicians to deliver highly accurate, minimally invasive, and patient-centered care for gastrointestinal and hepatopancreatobiliary disorders worldwide.
